# Cross-cultural adaptation and validation of the child perceptions questionnaire (CPQ_11–14_) among children in Lebanon

**DOI:** 10.1186/s12903-018-0482-x

**Published:** 2018-02-06

**Authors:** Adib Kassis, Nada El Osta, Stéphanie Tubert-Jeannin, Martine Hennequin, Lana El Osta, Joseph Ghoubril

**Affiliations:** 1 Department of Orthodontics, Saint-Joseph University, Beirut, Lebanon; 2 Department of Prosthodontics, Saint-Joseph University, Beirut, Lebanon; 3 University of Clermont Auvergne, EA 4847, Centre de Recherche en Odontologie Clinique, BP 10448, F-63000 Clermont-Ferrand, France; 4 Department of Public Health, Saint-Joseph University, Beirut, Lebanon; 5 CHU Clermont-Ferrand, Dental department, F-63000 Clermont-Ferrand, France

**Keywords:** Child perceptions questionnaire, Lebanon, Oral health, Psychometric, Child

## Abstract

**Background:**

The aim of this study was to assess the reliability, reproducibility, and convergent and discriminant validity of the Child Perceptions Questionnaire (CPQ)_11–14_ in a group of 11–14-year-old Lebanese children.

**Methods:**

This was a cross-sectional study. Children aged between 11 and 14 were recruited between March and June 2014 from five schools in Beirut and the surrounding region: a central public school and four private schools. Data were collected from self-administered questionnaires and a clinical intraoral examination was conducted. In addition to the CPQ_11–14_, the questionnaires included socio-demographic characteristics, questions about each child’s perception of oral and general health status and dental aesthetics, satisfaction with dental conditions and the need for dental treatment. The oral examination included the number of teeth affected by caries (D_3_ level), fillings, the number of missing teeth and an orthodontic assessment of malocclusion using the Dental Aesthetic Index (DAI).

**Results:**

The final number of children included was 693 and their mean age was 13.14 ± 0.82 years; 54.4% were boys. The mean CPQ score was 15.60 ± 14.55, range 0 to 110. Cronbach’s alpha of the global CPQ score was 0.880 and varied from 0.897 to 0.908. The reproducibility of the overall CPQ score was good, as was the CPQ score for each of the four domain scores (ICC > 0.682; *p* < 0.001). Higher CPQ scores were found for children with self-perceived poor general health (*p* < 0.001) or oral health (*p* < 0.001), unsatisfactory oral health (*p* < 0.001), need for dental treatment (*p* < 0.001) and perception of dental aesthetic problems (*p* < 0.001). Multiple linear regression models showed that the recruitment setting, the DMFT index and the DAI index were significantly associated with the CPQ global score (*p* = 0.004).

**Conclusion:**

The Lebanese version of the CPQ_11–14_ showed excellent psychometric properties and was able to distinguish children with different oral conditions. Additional longitudinal studies should be performed to test the properties of this questionnaire in clinical samples.

## Background

Adolescence is a critical period of life, associated with the specific social and psychological needs of children [1]. Oral health disorders such as malocclusion, caries and periodontal disease affect adolescents’ quality of life [2]. For instance, malocclusion, classified as a problem of growth and development of the maxilla and mandible during childhood and adolescence, may lead to functional and psychosocial disorders and affect the oral health related quality of life of adolescents [3–7]. Clinical variables were found to be of limited use for determining therapeutic needs and the concept of oral health related quality of life (OHRQoL) has been developed to quantify the extent to which oral health problems interfere with a patient’s daily life and well-being [8].

Several OHRQoL questionnaires have been promoted to assess children’s perceptions of oral health- related quality of life; one of the most commonly used is the Child Perception Questionnaire (CPQ). The CPQ was developed in Toronto to measure OHRQoL specifically for children [9]. Two questionnaires were designed for use in children aged 8–10 years (CPQ_8–10_) and 11–14 years (CPQ_11–14_) [10, 11]. The CPQ was later evaluated in many English-speaking countries and translated and validated in different cultural and linguistic situations [12–16]. It was translated into Arabic and validated in populations in Egypt, Syria and Saudi Arabia [17, 18]. The development of health research in several countries and multicultural aspects have increased the need for a cross-cultural adaptation of the CPQ_11–14_ to preserve validity through different cultures and to retain equivalency between the source and target languages [19]. Once the translated questionnaire has been adapted, its equivalence should be determined by testing its psychometric properties.

The effects of oral problems on quality of life have been integrated in epidemiological investigations and therapeutic programs to improve oral health around the world. These investigations did not exist in Lebanon and we could not find any surveys on OHRQoL in adolescents. The oral health conditions of the Lebanese child population were investigated using clinical health indicators like DMFT, the presence of malocclusion and dental fluorosis [20]. To our knowledge, oral health perception has never been assessed. Since clinical variables were found to be of limited use for determining therapeutic needs, subjective health indicators should be considered to assess the degree to which oral conditions affect social performance and lead to major changes in health behaviour [8].

The Arabic version of the CPQ validated in Saudi Arabia is available [17, 18] and its cultural adaptation for use in Lebanon is necessary due to divergences between health care systems, and between social and cultural aspects in other Arabic countries [21]. Lebanon is a country characterised by a free economy with no public dental health insurance system; dental care is therefore not accessible for children from low income families. However, free public oral health services are available to Saudi Arabians [22]. In addition, the cultural background of Arab countries and societies is deeply conservative and traditional. Lebanon has an Arab culture with western influences due to the emergence of various civilizations over thousands of years which has led to a culturally more liberal society [21]. The perception of quality of life and the ways in which health problems are expressed vary between diverse countries and cultures with similar languages [19]. These differences may alter the cultural equivalence and psychometric properties of the CPQ. It is therefore important to pretest and assess the validity of the Arabic version of CPQ_11–14_ among Lebanese children.

The aim of this study was therefore to adapt the CPQ to match Lebanese cultural specificities and to test its reliability, reproducibility and convergent validity_11–14_ in a group of 11–14-year-old Lebanese children. Moreover, the ability of the CPQ to distinguish between participants according to different oral health conditions (caries, malocclusion) was also evaluated.

## Methods

### Cross-cultural adaptation

The validated Arabic version of the CPQ_11–14_ was pre-tested on a sample of 25 Lebanese children aged between 11 and 14 years to ensure the cultural equivalency of the questionnaire and its suitability for use in Lebanon. Children were recruited from the Departments of Pedodontics and Orthodontics at Saint-Joseph University of Beirut and from a private clinic in Beirut. The meaning, comprehensibility and acceptability of the CPQ_11–14_ questions were studied. One modification was made to item 14 by adding between parentheses the translation in French of the word pipe, rarely used in Arabic in Lebanon. It should be noted that the Arabic version of the CPQ_11–14_ validated in Saudi Arabia does not include the item related to the difficulty encountered when playing a musical instrument [17, 18]. This item was not included in our study due to the very small number of Lebanese children who play wind instruments.

### Arabic version of the CPQ_11–14_ questionnaire

The Arabic version of the CPQ_11–14_ is composed of 36 items, worded negatively, divided into four health domains: oral symptoms (6 items), functional limitation (9 items), emotional well-being (9 items) and social well-being (12 items). Questions were asked about the frequency of events during the last three months. The response options were recorded: ‘Never’ = 0; ‘Once/twice’ = 1; ‘Sometimes’ = 2; ‘Often’ = 3; and ‘Everyday/almost every day’ = 4. The global CPQ_11–14_ score was obtained by summing all the item scores. The sum of the response code for questions in each subscale gave a total score for each domain [10]. Since there were 36 questions, the final score varied from 0 to 144, with a higher score indicating worse OHRQoL and a lower score indicating a better OHRQoL.

### Data collection

The protocol of this cross sectional study was approved by the Ethics Committee of Saint Joseph University of Beirut, (USJ-2014-9). Written informed consent was received from the parents and verbal consent was obtained from the children. Data were collected using self-administered questionnaires and intraoral clinical examinations.

#### Study population

Lebanese children aged between 11 and 14 years were recruited between March and June 2014 from five schools, a central public school and four private schools in Beirut and the surrounding area. Afterwards, the participants were randomly selected within the schools. The recruitment was carried out from three school years to comply with the age range of 11–14 years, using a randomization selection in each section from each year in both public and private schools. The students selected were invited to participate in the study. There were no specific guidelines with respect to appropriate sample power for testing the performance of the CPQ11–14 [9–12, 15, 17, 23, 24]. Consequently, the number of participants was arbitrarily set at 600, taking into account that the largest sample size used in a previous study was 561 [25].

#### Completion of the questionnaires

The questionnaires were completed during collective sessions in the classroom, in the presence of the teacher. The research investigator presented the aim of the study before individual oral examinations. He provided technical information to facilitate the completion of the questionnaires and collected the self-reported papers. No difficulties were reported concerning the comprehensibility of the CPQ.

In addition to the CPQ_11–14_ items, the questionnaire included items on socio-demographic characteristics (age, gender, recruitment setting) and questions on the child’s perception of his/her oral and general health and, in the case of dental aesthetics, his/her satisfaction with his/her dental conditions and feelings about the need for dental treatment. Participants were also asked if they were currently receiving or had previously received any orthodontic treatment.

#### Clinical data

Individual oral examinations were performed the same day in a medical examination room. One post-graduate student in Orthodontics conducted all the examinations according to WHO requirements [26]. Artificial light, equipment (gloves, mask and gauze pads) and pre-packaged sterilized instruments (single use mirror and periodontal probe) were used for the oral examinations. The number of teeth with carious lesions at D3 level according the Eckstrand classification [27], teeth with fillings and missing teeth were recorded. The DMFT Index was calculated for dental caries experience assessment according to the World Health Organization criteria [26]. An orthodontic assessment of malocclusion using the Dental Aesthetic Index (DAI) was also measured [26, 28] (Table 1). The DAI is a clinical index adopted by the World Health Organization to assess malocclusion in epidemiological studies. DAI can be used to indicate a need for orthodontic treatment; it combines clinical information and the aesthetic appearance of occlusion that indirectly assess social acceptability and relative dental appearance. It has proven to be a reliable, valid and easily applied index. It was developed to rank dental aesthetic and orthodontic treatment needs on a scale of social norms for socially acceptable dental appearance. The DAI rating is based on the measurement of ten occlusal traits related to appearance. In order to calculate the DAI score, each occlusal trait was multiplied by an appropriate weight and a constant (value 13) is added to the weighted sum. The scores vary from 13 (most acceptable) to 100 (least acceptable). The DAI scores can be categorised into four levels of malocclusion severity and treatment need: 13 to 25 (for normal or minor malocclusion with no or slight treatment need); 26 to 30 (definite malocclusion with elective orthodontic treatment); 31 to 35 (severe malocclusion with orthodontic treatment highly desirable) and 36 and over (for handicapping malocclusion with orthodontic treatment highly recommended). The DAI index was not calculated for those participants undergoing or who had completed orthodontic treatment [28].

**Table 1:** Dental Aesthetic Index items in order of recording

Aesthetic Index Components	Regression coefficients (Rounded weights)
Missing visible teeth (incisor, canine, or premolar teeth in maxillary and mandibular arches)	6
Crowding in the incisal segments (0 = no segment crowded; 1 = 1 segment crowded; 2 = 2 segments crowded)	1
Spacing in the incisal segments (0 = no spacing; 1 = 1 segment spaced; 2 = 2 segments spaced)	1
Midline diastema in mm	3
Largest anterior irregularity in mm (maxillary)	1
Largest anterior irregularity in mm (mandibular)	1
Anterior maxillary over-jet in mm	2
Anterior mandibular over-jet in mm	4
Vertical anterior open-bite in mm	4
Anterior-posterior molar relation (0 = Normal; 1 = 1/2cusp; 2 = Fullcusp)	3
Constant	13

### Statistical analyses

The statistical analyses were performed using the software SPSS for Windows, version 17.0, USA. The alpha error was set at 0.05. Reproducibility concerns the degree to which repeated measurements in stable persons provide similar answers. The test-retest reliability of the CPQ_11–14_ was assessed by repeating the administration of the CPQ_11–14_ two to three weeks after the first administration to 71 subjects who felt there had been no change in their oral health status since their first test. Reproducibility was measured by calculating the intra-class correlation coefficients (ICC) for the global score and each domain score with a two-way random effects model; ICC = 0.70 is recommended as a minimum standard for reproducibility [29].

Reliability or internal consistency is a measure of the extent to which items in a scale are correlated, thus measuring the same concept. The degree of internal consistency was determined using Cronbach’s alpha if an item was deleted. A low coefficient alpha would indicate that the items did not come from the same conceptual domain. A criterion of 0.6–0.9 is considered good reliability [29, 30].

The validation process of CPQ_11–14_ relies on the evaluation of concurrent validity, which examines a logical hypothesis by testing the index against a proxy measure of a similar concept [31]. It was hypothesised that subjects with higher OHRQoL scores would be less satisfied with their mouths, would report higher self-rated treatment need in the last three months and would have poorer self-rated oral and general health as well as poorer self-rated dental aesthetics. Concurrent validity was determined by establishing an association between the scores for the total CPQ_11–14_ scale and each domain with self-perception of general health, oral health, and dental aesthetics, satisfaction with oral health and self-reported need for dental treatment. Mann-Whitney and Kruskal Wallis tests were performed since the scores were not normally distributed.

Discriminant validity determines the ability of the CPQ to identify children with different oral health statuses. Discriminant validity was evaluated by comparing the CPQ_11–14_ global scores and each of the domain scores between different groups with objectively assessed dental status. It was hypothesised that patients with poor dental status (severe or handicapping malocclusion; > 4 decayed teeth; > 2 filled teeth; > 0 missing teeth; DMFT > 6) would have higher scores. The cut-off values for continuous clinical variables were chosen using the 75th percentiles. It was also hypothesised that scores could discriminate between participants with different socio-demographic characteristics such as age (11–12 years/13–14 years), sex and recruitment setting (public or private school). To test the discriminative properties, the overall CPQ_11–14_ and domain scores were studied according to the different malocclusion categories, number of decayed, filled and missing teeth, recruitment level, age categories and sex. Bivariable analyses were used followed by multivariable analyses after verifying the assumptions of multicollinearity and singularity of the independent parameters. Since the DMFT index is composed of the numbers of decayed, filled, and missing teeth, only the variable DMFT was included in the model. Finally, explanatory variables that were not related to scores in the bivariable analysis with *p*-values> 0.25 were not included in the multiple linear regression models.

## Results

### Description sample

Among the 764 school children eligible for the study, 43 (5.6%) did not return the signed consent form and 19 (2.5%) were absent from school during the data collection. The sample included 702 Lebanese children aged 11–14 years recruited from public and private schools. Three of them were excluded for behavioral problems during the completion of the questionnaire and six others did not cooperate enough for dental examination. Finally, 693 were included (response rate: 98.7%). The mean age was 13.14 ± 0.82 years and 54.4% were boys.

**Fig. 1:**
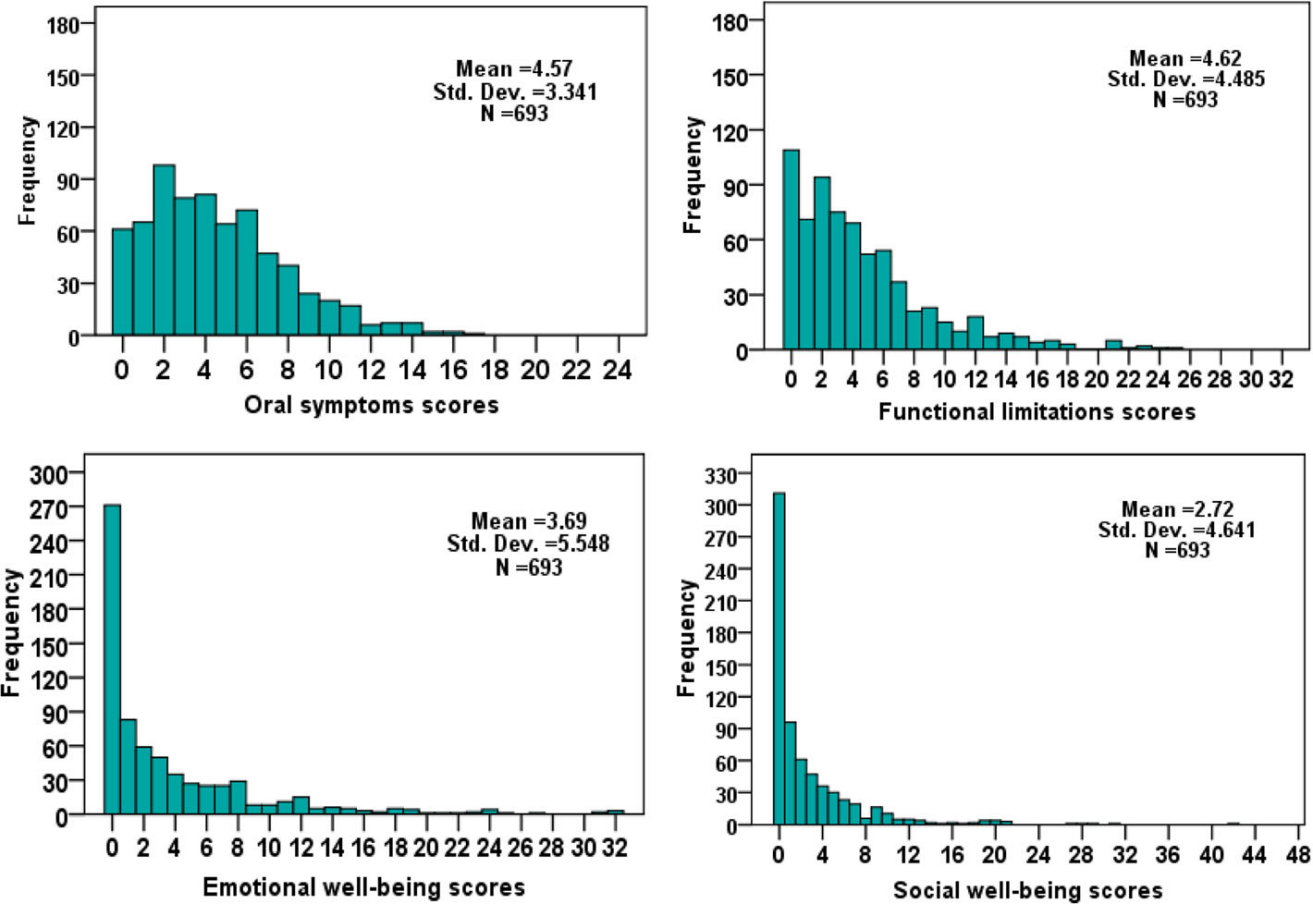
Distribution of the CPQ_11–14_ domain scores of a group of Lebanese children

**Fig. 2:**
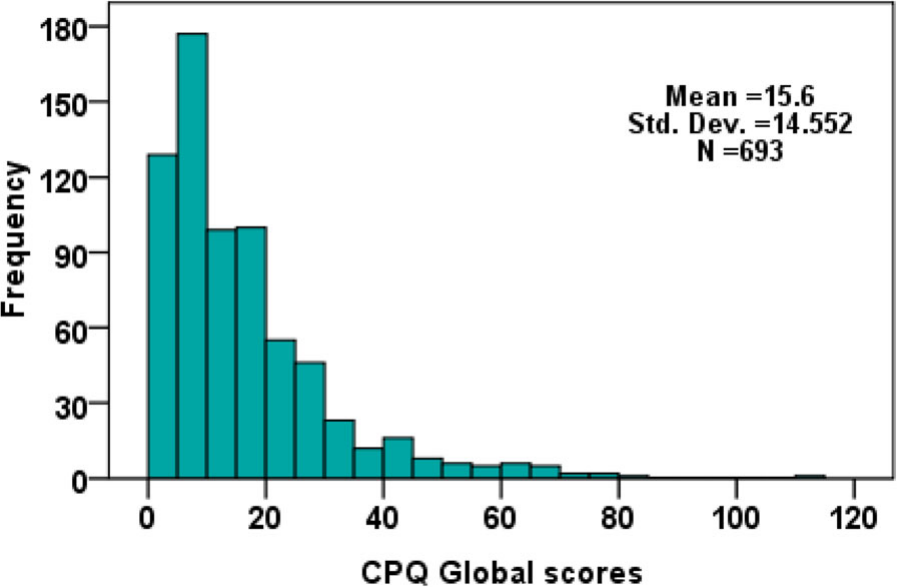
Distribution of the CPQ_11–14_ global scores of a group of Lebanese children

The mean CPQ_11–14_ score was 15.60 ± 14.55, range from 0 to 110. The distributions of CPQ11-14 for domain scores and global score are given in Figs. 1 and 2 respectively. According to the CPQ, 67.2% of the children had experienced oral symptoms in the previous three months, 63.3% had functional limitations, 37.1% had had emotional impacts and 28.0% had had social impacts. The responses to the different questions of CPQ _11–14_ are listed in Table 2.

**Table 2:** Proportion of children’s responses to the items of the Lebanese version of CPQ_11–14_

In the past 3 months	Never *N* (%)	Once or twice *N* (%)	Sometimes *N* (%)	Often *N* (%)	Everyday or almost every day *N* (%)
Dimension 1: Oral symptoms					
CPQ1 Pain in your teeth or mouth	353 (50.9%)	159 (22.9%)	138 (19.9%)	30 (4.3%)	13 (1.9%)
CPQ2. Bleeding gums	400 (57.7%)	128 (18.5%)	103 (14.9%)	30 (4.3%)	32 (4.6%)
CPQ3. Sore spots	374 (54.0%)	182 (26.3%)	103 (14.9%)	29 (4.2%)	5 (.7%)
CPQ4. Bad breath	436 (62.9%)	111 (16.0%)	95 (13.7%)	34 (4.9%)	17 (2.5%)
CPQ5. Food stuck in or between your teeth	251 (36.2%)	184 (26.6%)	166 (24.0%)	45 (6.5%)	47 (6.8%)
CPQ6. Food stuck in the top of your mouth	549 (79.2%)	88 (12.7%)	32 (4.6%)	16 (2.3%)	8 (1.2%)
Dimension 2: Functional limitations					
CPQ7. Breathed through your mouth	256 (36.9%)	145 (20.9%)	160 (23.1%)	59 (8.5%)	73 (10.5%)
CPQ8. Took longer than others to eat a meal	535 (77.2%)	89 (12.8%)	38 (5.5%)	14 (2.0%)	17 (2.5%)
CPQ9. Had Have trouble sleeping	552 (79.7%)	82 (11.8%)	41 (5.9%)	14 (2.0%)	4 (.6%)
CPQ10. Difficult to bite or chew food like apples, corn on the cob or steak	472 (68.1%)	94 (13.6%)	79 (11.4%)	36 (5.2%)	12 (1.7%)
CPQ11. Difficult to open your mouth wide	576 (83.1%)	59 (8.5%)	34 (4.9%)	12 (1.7%)	12 (1.7%)
CPQ12. Difficult to say any words	595 (85.9%)	44 (6.3%)	27 (3.9%)	17 (2.5%)	10 (1.4%)
CPQ13. Difficult to eat foods you would like to eat	535 (77.2%)	74 (10.7%)	49 (7.1%)	28 (4.0%)	7 (1.0%)
CPQ14. Difficult to drink with a straw	630 (90.9%)	29 (4.2%)	19 (2.7%)	10 (1.4%)	5 (.7%)
CPQ15. Difficult to drink or eat hot or cold foods	390 (56.3%)	143 (20.6%)	90 (13.0%)	42 (6.1%)	28 (4.0%)
Dimension 3: Emotional well-being					
CPQ16. Felt irritable or frustrated	563 (81.2%)	70 (10.1%)	29 (4.2%)	19 (2.7%)	12 (1.7%)
CPQ17. Felt unsure of yourself	570 (82.3%)	56 (8.1%)	36 (5.2%)	18 (2.6%)	13 (1.9%)
CPQ18. Felt shy or embarrassed	507 (73.2%)	96 (13.9%)	49 (7.1%)	22 (3.2%)	19 (2.7%)
CPQ19. Concerned about what other people think about your teeth or mouth	490 (70.7%)	104 (15.0%)	59 (8.5%)	25 (3.6%)	15 (2.2%)
CPQ20. Worried that you are not as good-looking as others	521 (75.2%)	83 (12.0%)	57 (8.2%)	17 (2.5%)	15 (2.2%)
CPQ21. Felt upset	457 (65.9%)	120 (17.3%)	73 (10.5%)	27 (3.9%)	16 (2.3%)
CPQ22. Felt nervous or afraid	568 (82.0%)	69 (10.0%)	33 (4.8%)	16 (2.3%)	7 (1.0%)
CPQ23. Worried that you are not as healthy as others	564 (81.4%)	70 (10.1%)	37 (5.3%)	11 (1.6%)	11 (1.6%)
CPQ24. Worried that you are different from others	548 (79.0%)	83 (12.0%)	37 (5.3%)	10 (1.4%)	15 (2.2%)
Dimension 4: Social well-being					
CPQ25. Missed school because of your teeth or mouth	586 (84.6%)	75 (10.8%)	23 (3.3%)	9 (1.3%)	0 (0.0%)
CPQ26. Had problems paying attention in school	549 (79.2%)	86 (12.4%)	40 (5.8%)	14 (2.0%)	4 (.6%)
CPQ27. Had difficulty doing your homework	589 (85.0%)	58 (8.4%)	28 (4.0%)	14 (2.0%)	4 (.6%)
CPQ28. Did not want to speak or read out loud in class	566 (81.7%)	80 (11.5%)	33 (4.8%)	11 (1.6%)	3 (.4%)
CPQ29. Stayed away from sports and club activities	641 (92.5%)	34 (4.9%)	10 (1.4%)	5 (.7%)	3 (.4%)
CPQ30. Did not want to talk to other children	620 (89.5%)	50 (7.2%)	16 (2.3%)	6 (.9%)	1 (.1%)
CPQ31. Avoid smiling or laughing with other children	524 (75.6%)	95 (13.7%)	46 (6.6%)	18 (2.6%)	10 (1.4%)
CPQ33. Did not want to spend time with other children	642 (92.6%)	22 (3.2%)	18 (2.6%)	8 (1.2%)	3 (.4%)
CPQ34. Argued with other children or your family	613 (88.5%)	46 (6.6%)	17 (2.5%)	12 (1.7%)	5 (.7%)
CPQ35. Other children teased you or called you names	605 (87.3%)	47 (6.8%)	24 (3.5%)	12 (1.7%)	5 (.7%)
CPQ36. Other children made you feel left out	640 (92.3%)	34 (4.9%)	12 (1.7%)	5 (.7%)	2 (.3%)
CPQ37. Other children asked you questions about your teeth or mouth	551(79.5%)	87(12.6%)	34(4.9%)	15(2.2%)	6(.9%)

Oral conditions were frequent in our study population; 61.9% of the children had at least one untreated carious lesion, 8% had had or were undergoing orthodontic treatment, 20.3% had definite malocclusion with elective orthodontic treatment and 15.9% had severe or handicapping malocclusion with orthodontic treatment highly recommended.

### Reliability

Cronbach’s alpha of the global CPQ_11–14_ score was 0.880 and varied from 0.897 to 0.908 when item 21 (felt upset) and item 7 (Breathed through your mouth) were deleted respectively. Cronbach’s alpha for each of the four domains were as follows: Oral symptoms = 0.530; Functional limitations = 0.697; Emotional Well-Being = 0.874; Social Well-Being = 0.848.

### Reproducibility

The test–retest reliability of the overall CPQ_11–14_ was high (ICC = 0.711; *p* < 0.001), likewise for the domains of oral symptoms (ICC = 0.700; *p* < 0.001), functional limitations (ICC = 0.702; *p* < 0.001), emotional well-being (ICC = 0.722; *p* < 0.001) and social well-being (ICC = 0.682; *p* < 0.001).

### Concurrent validity

Higher CPQ scores were found in children who perceived their general or oral health to be poor or very poor (for both, *p* < 0.001), who reported a need for dental treatment (*p* < 0.001) and who felt they had dental aesthetic problems (*p* < 0.001) (Tables 3 and 4).

**Table 3:** Concurrent validity of the Lebanese version of the CPQ_11–14_

		Dimension 1- Oral Symptoms	Dimension 2- Functional limitations	Dimension 3- Emotional well being	Dimension 4- Social well being	Global score
Self perception of general health	Poor/very poor (*n* = 40)	6.40 ± 3.79	7.55 ± 5.34	7.18 ± 8.41	5.35 ± 7.21	26.48 ± 18.90
	Moderate (*n* = 118)	6.44 ± 3.58	7.23 ± 5.19	6.99 ± 8.08	5.69 ± 7.20	26.36 ± 19.85
	Good/Very good (*n* = 533)	4.01 ± 3.05	3.82 ± 3.92	2.70 ± 4.02	1.87 ± 3.10	12.40 ± 10.85
	*Sig*	*< 0.001*	*< 0.001*	*< 0.001*	*< 0.001*	*< 0.001*
Self perception of oral health	Poor/very poor (*n* = 84)	7.02 ± 3.86	8.21 ± 5.80	9.73 ± 8.69	7.36 ± 8.05	32.32 ± 20.68
	Moderate (*n* = 219)	5.11 ± 3.07	5.22 ± 4.56	4.23 ± 4.99	3.06 ± 3.93	17.62 ± 12.68
	Good/Very good (*n* = 388)	3.72 ± 3.05	3.50 ± 3.57	2.08 ± 3.76	1.53 ± 3.12	10.83 ± 10.51
	*Sig*	*< 0.001*	*< 0.001*	*< 0.001*	*< 0.001*	*< 0.001*
Satisfaction with oral health	Not satisfied (*n* = 112)	6.01 ± 3.70	7.12 ± 5.80	7.71 ± 7.86	5.94 ± 7.32	26.77 ± 19.73
	Moderately (*n* = 160)	5.38 ± 3.12	5.02 ± 4.54	5.12 ± 5.88	3.44 ± 4.61	18.96 ± 14.47
	Very satisfied (*n* = 419)	3.87 ± 3.14	3.80 ± 3.75	2.07 ± 3.69	1.59 ± 3.02	11.32 ± 10.56
	*Sig*	*< 0.001*	*< 0.001*	*< 0.001*	*< 0.001*	*< 0.001*
Self reported need for dental treatment	Yes (n = 419)	5.23 ± 3.41	5.29 ± 4.77	4.92 ± 6.42	3.62 ± 5.44	19.06 ± 16.12
	No (*n* = 272)	3.54 ± 2.97	3.58 ± 3.79	1.80 ± 3.02	1.34 ± 2.49	10.26 ± 9.64
	*Sig*	*< 0.001*	*< 0.001*	*< 0.001*	*< 0.001*	*< 0.001*

**Table 4:** Association between the CPQ_11–14_ scores and perception of dental aesthetics

		Dimension 1- Oral Symptoms	Dimension 2- Functional limitations	Dimension 3- Emotional well being	Dimension 4- Social well being	Global score
How much do you like the look of your teeth	Never (*n* = 100)	6.01 ± 3.82	6.95 ± 5.50	8.64 ± 7.97	6.07 ± 7.41	27.67 ± 19.94
	Moderately (*n* = 349)	4.73 ± 3.28	4.78 ± 4.57	3.81 ± 5.18	2.92 ± 4.38	16.24 ± 13.87
	Very much (*n* = 242)	3.74 ± 2.99	3.42 ± 3.37	1.46 ± 2.90	1.06 ± 2.02	9.67 ± 8.41
	*Sig*	*< 0.001*	*< 0.001*	*< 0.001*	*< 0.001*	*< 0.001*
Your teeth are	Less beautiful than others (*n* = 90)	6.74 ± 4.11	6.74 ± 5.45	8.83 ± 8.17	6.12 ± 7.06	28.44 ± 19.52
	Like others (*n* = 504)	4.28 ± 2.96	4.29 ± 4.10	2.94 ± 4.48	2.11 ± 3.63	13.62 ± 11.98
	More beautiful than others (*n* = 96)	4.06 ± 3.70	4.41 ± 4.94	2.81 ± 5.11	2.81 ± 5.24	14.09 ± 15.63
	*Sig*	*< 0.001*	*< 0.001*	*< 0.001*	*< 0.001*	*< 0.001*
You think your teeth are	Less aligned than others (*n* = 186)	5.47 ± 3.32	5.88 ± 4.81	5.97 ± 6.98	4.20 ± 6.17	21.52 ± 17.37
	Aligned as others (*n* = 431)	4.13 ± 3.19	4.00 ± 4.19	2.80 ± 4.55	2.07 ± 3.51	12.99 ± 12.19
	More aligned than others (*n* = 73)	4.92 ± 3.79	5.12 ± 4.69	3.16 ± 5.31	2.86 ± 5.21	16.07 ± 15.44
	*Sig*	*< 0.001*	*< 0.001*	*< 0.001*	*< 0.001*	*< 0.001*

### Discriminant validity

The overall CPQ score and the four dimension scores were not significantly associated with the gender (*p* = 0.945) and the age of the participants (*p* = 0.361). Children recruited from public schools experienced higher oral health impacts (Table 5).

**Table 5:** Discriminant validity of CPQ_11–14_ scores in bivariable analysis

		Dimension 1- Oral Symptoms	Dimension 2- Functional limitations	Dimension 3- Emotional well being	Dimension 4- Social well being	Global score
Gender	Boy (*n* = 377)	4.67 ± 3.44	4.59 ± 4.22	3.62 ± 5.35	2.75 ± 4.63	15.64 ± 14.02
	Girl (*n* = 316)	4.45 ± 3.23	4.65 ± 4.79	3.77 ± 5.78	2.69 ± 4.66	15.56 ± 15.18
	*Sig*	*0.390*	*0.860*	*0.725*	*0.851*	*0.945*
Age categories	11–12 years (n = 160)	4.74 ± 3.64	5.06 ± 4.71	4.11 ± 5.23	2.62 ± 4.18	16.52 ± 14.50
	13–14 years (n = 533)	4.52 ± 3.25	4.49 ± 4.41	3.57 ± 5.64	2.75 ± 4.77	15.32 ± 14.57
	*Sig*	*0.450*	*0.160*	*0.281*	*0.746*	*0.361*
Recruitment level	Public (*n* = 307)	4.60 ± 3.60	5.21 ± 5.08	4.68 ± 6.44	3.05 ± 4.81	17.55 ± 16.24
	Private (*n* = 386)	4.54 ± 3.13	4.15 ± 3.90	2.90 ± 4.58	2.46 ± 4.50	14.05 ± 12.87
	*Sig*	*0.811*	*0.002*	*< 0.001*	*0.099*	*0.002*
Malocclusion category	None/Minor (*n* = 387)	4.26 ± 3.08	4.13 ± 3.84	2.72 ± 3.95	1.88 ± 3.12	12.99 ± 10.58
	Definite (*n* = 141)	4.82 ± 3.69	4.78 ± 4.61	4.13 ± 5.49	3.06 ± 4.88	16.79 ± 15.37
	Severe/handicapping (*n* = 110)	5.06 ± 3.52	5.73 ± 6.15	6.80 ± 8.73	4.72 ± 7.41	22.31 ± 22.17
	*Sig*	*0.037*	*0.004*	*< 0.001*	*< 0.001*	*< 0.001*
Number of decayed teeth	≤4 (*n* = 570)	4.41 ± 3.30	4.48 ± 4.36	3.34 ± 5.26	2.47 ± 4.03	14.70 ± 13.42
	> 4 (*n* = 119)	5.20 ± 3.35	5.16 ± 4.99	5.24 ± 6.47	3.88 ± 6.77	19.49 ± 18.47
	*Sig.*	*0.019*	*0.131*	*0.001*	*0.003*	*0.001*
Number of filled teeth	≤ 2 (*n* = 542)	4.63 ± 3.37	4.70 ± 4.50	3.86 ± 5.67	2.81 ± 4.54	16.00 ± 14.54
	> 2 (*n* = 147)	4.26 ± 3.15	4.22 ± 4.40	2.95 ± 4.93	2.38 ± 5.01	13.80 ± 14.37
	*Sig.*	*0.230*	*0.250*	*0.074*	*0.325*	*0.104*
Missing teeth	≤0 (*n* = 666)	4.54 ± 3.33	4.53 ± 4.46	3.48 ± 5.28	2.58 ± 4.43	15.13 ± 14.17
	> 0 (*n* = 23)	4.78 ± 3.16	6.43 ± 4.87	9.17 ± 8.94	6.61 ± 7.99	27.00 ± 19.38
	*Sig.*	*0.733*	*0.045*	*< 0.001*	*< 0.001*	*< 0.001*
DMFT	≤6 (*n* = 608)	4.44 ± 3.29	4.47 ± 4.41	3.47 ± 5.38	2.52 ± 4.14	14.91 ± 13.77
	> 6 (*n* = 81)	5.36 ± 3.44	5.51 ± 4.88	5.12 ± 6.39	4.16 ± 7.28	20.15 ± 18.67
	*Sig.*	*0.020*	*0.051*	*0.012*	*0.003*	*0.002*

Participants with severe or handicapping malocclusion presented more OHRQoL impacts when compared with those with minor malocclusion categories (*p* < 0.05). Moreover, children with > 4 decayed teeth and > 0 missing teeth more frequently had higher CPQ scores (*p* < 0.05). However, the number of filled teeth was not related to the CPQ scores.

Multiple linear regression models were analysed using the following explanatory variables: age, sex, recruitment setting, malocclusion categories and number of decayed teeth (> 4), number of filled teeth (> 2) and number of missing teeth (> 0) (Table 6). For caries experience, only the variable DMFT was included in the model.

**Table 6:** Discriminant validity of CPQ_11–14_ scores in multiple linear regression analyses

	Unstandardized Coefficients		Standardized Coefficients	t	Sig.	Correlations
	B	Std. Error	Beta			Part
Global score						
Age	−0.214	1.380	−0.006	−0.155	0.877	−0.006
Recruitment level	−3.409	1.177	− 0.116	−2.897	0.004	−0.111
DMFT	5.089	1.721	0.114	2.957	0.003	0.113
DAI index	4.316	0.734	0.226	5.880	0.000	0.224
Oral symptoms						
Age	−0.201	0.321	−0.026	− 0.626	0.531	− 0.025
Recruitment level	−0.005	0.274	0.000	−0.018	0.985	0.000
DMFT	0.992	0.400	0.098	2.479	0.013	0.098
DAI index	0.442	0.171	0.103	2.594	0.010	0.102
Functional limitation						
Age	−0.224	0.434	−0.021	−0.517	0.605	−0.020
Recruitment level	−1.192	0.370	−0.131	−3.224	0.001	−0.126
DMFT	0.952	0.541	0.069	1.761	0.079	0.069
DAI index	0.704	0.231	0.120	3.052	0.002	0.119
Emotional well-being						
Age	−0.060	0.521	−0.005	−0.114	0.909	−0.004
Recruitment level	−1.582	0.445	−0.141	−3.558	0.000	−0.135
DMFT	1.540	0.650	0.090	2.369	0.018	0.090
DAI index	1.832	0.277	0.251	6.608	0.000	0.250
Social well-being						
Age	0.271	0.440	0.025	0.615	0.539	0.024
Recruitment level	−0.631	0.376	−0.068	−1.680	0.094	−0.065
DMFT	1.605	0.549	0.113	2.922	0.004	0.112
DAI index	1.338	0.234	0.221	5.712	0.000	0.220

The multivariable analyses showed that the recruitment setting was significantly associated with the CPQ global score (*p* = 0.004), functional limitations (*p* = 0.001) and emotional well-being (*p* < 0.001).

The DMFT index was significantly associated with the CPQ global score (*p* = 0.003), oral symptoms (*p* = 0.013), emotional well-being s (*p* = 0.018) and social well-being (*p* = 0.004).

The DAI index was significantly associated with the CPQ global score (*p* < 0.001), oral symptoms (*p* < 0.001), functional limitations (*p* < 0.001), emotional well-being (*p* < 0.001) and social well-being (*p* < 0.001).

## Discussion

This study showed that the psychometric properties of the Lebanese version of the CPQ11_− 14_ were suitable in a group of Lebanese children aged between 11 and 14 years. The reliability of the CPQ was appropriate since Cronbach’s alpha for the overall CPQ score and each of the domain scores was higher than 0.5 and demonstrated the homogeneity of the items, as has already been verified in previous Brazilian, Canadian, Saudi Arabian, Italian and English versions [7, 9–11, 16, 17, 25]. The coefficient was higher for emotional well-being (0.874) and lower for the oral symptoms domains (0.530) due to the item 6 “Food stuck in the top of your mouth” that correlates less with the other items of the same domain. When 71 subjects re-filled the questionnaire two to three weeks after it was first administered, given that the patients’ clinical status had not changed, the test-retest findings revealed very good reproducibility for the overall CPQ_11–14_ and each of the underlying subscales (ICC > 0.7). Values higher than or equal to 0.7 were considered acceptable [29]. This finding was similar to that reported in the previous Canadian, Brazilian and Saudi Arabian studies [10, 12, 17, 18]. The assessment of concurrent validity was based on the support of theoretical relationships between the CPQ_11–14_ and another questionnaire that assessed similar constructs. Concurrent validity was carried out with the expected associations between the CPQ_11–14_ scale scores and the global rating of oral health, general health, self-reported need for orthodontic treatment and self-perception of aesthetics and alignment of teeth. The analysis confirmed what was established: that a higher CPQ score and subscale scores for each of the four domains were associated with poorer self-perceived oral and general health, poorer self-perceived alignment of teeth and a need for orthodontic treatment. The concurrent validity of the CPQ11–14 has previously been demonstrated in several validation studies [9, 16, 17].

Dental caries assessment and need for orthodontic treatment were used to assess the discriminant validity of the CPQ1_11–14_ and to compare our findings with other studies [11, 12, 18, 23, 25]. Dental caries and malocclusion are among the most commonly studied oral diseases that interfere with the normal functioning of an individual’s life, causing pain, chewing difficulties and lack of sleep which affect learning and growth [1, 2, 5–7, 32]. The Lebanese version of the CPQ_11–14_ was able to discriminate between Lebanese children according to recruitment level, DMFT index, and malocclusion status for the overall score and some of the subscale scores. However, the gender and age of the participants were not associated with the CPQ score, in contrast with other studies that found gender differences; girls reporting poorer OHRQoL, except for the oral symptoms domain which was higher for boys [33, 34]. Need for prthodontic treatment affects children’s quality of life [5, 35, 36]. This was certainly the case with the CPQ11–14 scores, with a clear ascending score demonstrated across ascending categories of orthodontic treatment need. Children with a higher DAI index, indicating severe or handicapping malocclusion, showed a higher CPQ_11–14_ global score. Additionally, participants with severe or handicapping malocclusion suffered from more functional limitations and oral symptoms and lower emotional and social well-being than participants with normal or minor malocclusion. Mal-occluded teeth can cause psychosocial problems related to impaired dento-facial aesthetics, disturbances of oral function such as mastication, swallowing and speech and greater susceptibility to decay and periodontal disease, and may be important motivating factors for initiating orthodontic treatment [1, 4, 7]. The present results were consistent with other studies which explored how malocclusion affects children’s quality of life [32, 35]. However, this was not the case in a Saudi Arabian study, since malocclusion was not associated with oral symptoms, functional limitations and emotional well-being; also, DMFT was not related to the overall score and subscale scores. These discrepancies may justify the fact that quality of life and the ways in which oral health problems are expressed can be affected by cultural aspects, social influences and beliefs between Lebanese and Saudi Arabian children [17, 19]. Discriminant validity testing on known groups revealed higher OHRQoL scores for children with an elevated DMFT index and a greater impact on oral symptoms, and emotional and social well-being. It should be noted that caries experience and the presence of restorations were important discriminating factors related to the total score and the four domains separately in certain validation studies [10]. The DMFT index indicates the severity of caries experience and its relation to the care needs of a population. Its relationship with OHRQoL can help policymakers to better understand how to devise a national dental policy specifically designed to meet people’s needs rather than to apply the normative criteria of dentists.

It was previously suggested that clinical indicators like caries experience and malocclusion are not the only factors associated with OHRQoL [37, 38]; account must also be taken of social background and family environmental variables that affect children’s daily life [1, 14, 33, 37]. Lower OHRQoL levels were observed in children who attend low cost public schools compared with those attending more expensive private schools [38, 39]. The OHRQoL revealed that Lebanese children attending public schools had higher CPQ_11–14_ scores than others. This was in agreement with several cross-sectional studies that showed that pupils from public schools (free of payment) came from social backgrounds different from those of children attending private schools. These findings underpin the evidence that oral health perception can be affected by social influences.

The results of this study should be interpreted by taking into account certain limitations relating to the lack of representativeness of the sample. Participants were recruited in five schools in Beirut and its surrounding area. The results of this survey are therefore not applicable to schoolchildren in other cities in the country and so cannot be used to describe the oral health of all Lebanese schoolchildren.

The strength of the study lies in the very high (98.7%) response rate of the children and in the large number of participants, since there were no specific guidelines with respect to the sample power appropriate for testing the performance of the CPQ_11–14_. A sample size of 693 schoolchildren was proposed which was higher than that used in previous CPQ validation studies _11–14_. The highest sample size was 561 schoolchildren in a study conducted in Italy [25], then 457 in Malaysia [23] and 468 schoolchildren in Australia [24], while the lowest was 89 in a study conducted in the United Kingdom [40]. Only three previous studies used population-based random samples of schoolchildren [11, 13, 24], while others used convenience samples of either children attending public schools [14, 15, 40], or children attending paediatric clinics [9, 10, 16, 17].

## Conclusion

The Lebanese version of the CPQ_11–14_ showed excellent psychometric properties and was able to distinguish children with different oral conditions. It also indicated that the impact of child oral conditions on functional and psychosocial well-being is considerable and that children are able to give psychometrically acceptable accounts of that impact. Further longitudinal studies are required to investigate its evaluative properties and its utility as a measure of clinical outcome in clinical trials.

## Abbreviations

CPQ_11–14_: Child Perception Questionnaire
DAI: Dental Aesthetic Index
DMFT: Decayed, missing and filled teeth
OHRQoL: Oral Health Related Quality of Life
